# 

**Published:** 1895-04-06

**Authors:** 


CONTENTS.

Drink and Accidents - 1

The Position of "The Hospital" - 2

Modern Medico-Psychology and Psychiatry. By T. S. Clouston, M.D.—Conditions of Mental Stupor - 3

Medical Progress and Hospital Clinics:

Suppurative Inflammation of Joints in Children - 7

Progress in Medicine.—Diseases of the Circulatory System - 9

Progress in Surgery.—Genito-Urinary Surgery - 10

Therapeutic Notes.—Leech Extracts and Thrombosis—Unusual Effects of Hyoscine - 12

New Appliances and Things Medical - 12

Modern Sociology.—The Training of the Hands - 4

Annotations.—Cheap Dispensing—The Annual Meeting—Mushrooms and Manure-heaps - 5

Notes and News - 6

The Institutional Workshop:

Hospital Construction.—The Manchester Royal Infirmary - 13

The Story of the Insane from Year to Year - 14

Practical Points - 15

Hospital Festivals and Meetings - 15

Scraps and Gleanings - 16

The Book World of Medicine and Science - 17

EXTRA SUPPLEMENT.—THE NURSING MIRROR.

News from the Nursing World.—Royalty in North London—At Paddington Green—Bad Times for Babies—The S.P.C.K. Medical Mission—A Little Dispensing—Society of Trained Masseuses—Better Late than Never—Probationers at Bristol—Newcastle Nurses—The Sick Poor at Whitehaven—Nottingham—Inadequate Remuneration—District Nursing in Wales—Short Items - i

Elementary Anatomy and Surgery for Nurses. By W. McAdam Eccles, M.B., M.S., F.R.C.S. - iii

Nursing in France - iv

Notes and Queries - iv

Asylum News and Nursing - v

A Great Movement: The Nurses Co-operation. II. - vi

Wants and Workers - vi

Junius S. Morgan Benevolent Fund - vii

Everybody's Opinion - viii

Novelties for Nurses - viii

Appointments - viii

